# 11th Intelligent Systems for Molecular Biology 2003 (ISMB 2003)

**DOI:** 10.1002/cfg.336

**Published:** 2003-12

**Authors:** Catherine A. Abbott

**Affiliations:** School of Biological Sciences Flinders University GPO BOX 2100 Adelaide SA Australia

## Abstract

This report profiles the keynote talks given at ISMB03 in Brisbane, Australia by Ron Shamir, David Haussler, John Mattick, Yoshihide Hayashizaki, Sydney Brenner, the Overton Prize winner, Jim Kent, and the ISCB Senior Accomplishment Awardee, David Sankov.


**Comparative and Functional Genomics**
*Comp Funct Genom* 2003; **4**: 654–659.
Published online in Wiley InterScience (www.interscience.wiley.com). **DOI:** 10.1002/cfg.336

Meeting Review

### 11th Intelligent Systems for Molecular Biology 2003 (ISMB 2003)

Brisbane Convention Centre, Brisbane, Australia, 29 June–3 July 2003

Catherine A. Abbott*
*School of Biological Sciences, Flinders University, Adelaide, SA, Australia*

*Correspondence to: Catherine A. Abbott, School of Biological Sciences, Flinders University, GPO BOX 2100, Adelaide, SA, Australia. E-mail: cathy.abbott@flinders.edu.au*

Received: 22 September 2003
Revised: 25 September 2003
Accepted: 29 September 2003

### Abstract

**This report profiles the keynote talks given at ISMB03 in Brisbane, Australia by Ron Shamir, David Haussler, John Mattick, Yoshihide Hayashizaki, Sydney Brenner, the Overton Prize winner, Jim Kent, and the ISCB Senior Accomplishment Awardee, David Sankov. Copyright © 2003 John Wiley & Sons, Ltd.**

### Introduction

This year for the first time the annual meeting of the International Society of Computational Biology (ISCB) was held in the Southern hemisphere. The 11th International Conference on Intelligent Systems in Molecular Biology (ISMB2003) came 'down under' and was held at the Brisbane Convention Centre, Brisbane, Australia, 29 June–3 July 2003. Unfortunately, the promised great Aussie sun did not come out as much as was expected but the programming committee delivered a stimulating program where the 'bio' was once again returned to bioinformatics.

As is traditional at ISMB, the meeting was preceded by Special Interest Group (SIGS) satellite meetings and tutorials. These SIGS were well attended, with the numbers of participants ranging from 98 for the Bioinformatics Open Source Conference, 100 for Biopathways, 51 for Bio-Ontologies, 28 for WEB03, and 42 for Text Mining (BioLINK). The tutorials were popular, as usual, with over 500 ISMB registrants taking part in the 15 tutorials offered. These were held on traditional topics, such as 'Molecular Phylogenetics and Evolutionary Analysis' and 'Molecular Modelling: building a 3D protein structure from its sequence', and on more cutting-edge topics such as 'Bioethics for Bioinformaticists', 'Artificial Intelligence and Machine Learning Techniques for Bioinformatics' and 'Data Warehousing in Molecular Biology'.

The official meeting began on Monday with a warm welcome by the co-chairs of the meeting, Gene Myers and Mark Ragan, to the 919 registrants from 42 different countries. This review covers the keynote and prize-winning talks that were delivered across the 4 days of the meeting. All other talks are published as papers in the journal *Bioinformatics* (**http://bioinformatics.oupjournals.org/**). Notable speakers at ISMB 2003 included: Sydney Brenner (one of three recipients of the 2002 Nobel Prize in Medicine, founder of the Molecular Sciences Institute and Distinguished Research Professor at the Salk Institute); David Haussler (Howard Hughes Medical Institute Investigator and Professor of Computer and Information Sciences at the University of California, Santa Cruz); Yoshihide Hayashizaki (Project Director of the Genome Exploration Research Group at the Genomic Sciences Center at RIKEN); John Mattick (Director of

Copyright © 2003 John Wiley & Sons, Ltd.

the Institute for Molecular Bioscience, University of Queensland); Ron Shamir (Professor of Computer Science at Tel-Aviv University); and Michael Waterman (Professor of Mathematics, Computer Science and Biological Science at the University of Southern California).

This year's ISMB focused more on the use of bioinformatics and computational biology to analyse entire biological systems and there was less emphasis on individual standalone problems, such as microarray analysis, building phylogenetic trees, motif- and gene-finding algorithms. Papers and keynotes at the conference were divided into seven major themes: (1) phylogeny and genome rearrangements; (2) expression arrays and networks; (3) predicting clinical outcomes; (4) protein clustering, alignment and patterns; (5) transcription motifs and modules; (6) structure and hidden Markov models; and (7) text mining and high-throughput methods; with an additional session for short papers.

With the sequences of over 1000 genomes now completed, including those of human [13] and mouse [20], the meeting opened with a thought-provoking session on phylogeny and genome rearrangements, which included two keynote speakers.

**David Haussler** (**University of California; Santa Cruz**; **http://www.cse.ucsc.edu/~haussler**) gave a fascinating presentation on 'Identifying functional elements in the human genome by tracing the evolutionary history of the bases: a key challenge for comparative genomics'. He outlined the principles of using evolution to find genes and other functional elements [19]. Although 75 million years of evolution separate mice and humans, comparing the genomes of the two species provides a crude way of finding regions of functional significance. This type of approach is noisy and, while difficult, it is possible to separate the noise from the useful information using a calibration point. At least half of the human genome consists of relics of retrotransposons, i.e. half of our genome is the rotting carcasses of the selfish DNA that has inserted itself into our DNA over the years. Comparative genomics will allow us to identify functional elements and, as more species are sequenced, there will be an increase in our power to detect conserved elements. His group has enhanced these studies by combining hidden Markov and phylogenetic models to create new ways of modelling molecular evolution [17].

One of the grand challenges of human molecular evolution is to reconstruct the evolutionary history of each base in the human genome. Great sums of money were spent on getting a draft sequence of the human genome, so we now need to use this information fruitfully. Genome browsers, such as that at UCSC, will become more than archives of data — they will be used as microscopes that can be used to interpret and discover new things about human genes. The overall goal of future browser applications is to link gene position to functional information, so that the browser can be used as an engine for discovery — a microscope searching the genome for new and exciting discoveries. These thoughts were expanded upon later in the meeting in the Overton Prize address by Jim Kent.

The second keynote address in this session was **John Mattick's (University of Queensland**; **http://www.imb.uq.edu.au/mattick.html**), 'Programming of the autopoietic development of complex organisms: the hidden layer of non-coding RNA'. His presentation generated enormous excitement in the audience and attracted an incredible amount of interest in the form of questions afterwards.

The number of protein-coding genes does not scale strongly with the complexity of an organism. The fly and worm genomes contain 14 000–19 000 protein-coding genes, two to three times more than yeast at ~6000, but this is not much less than the number of mammalian protein-coding genes. Moreover, the genome of the inanimate plant, rice, has more genes than that of humans. The 98% of non-coding sequence in the human genome actually has a tighter correlation with human complexity. Most of the genetic variation between organisms lies in these non-coding regions, indeed only 1% of coding genes between mouse and human are different. Mattick gave a compelling argument that non-coding RNA is the genetic basis of human complexity and variation [14,15]. There are enormous numbers of non-coding RNA genes in the mammalian genome, which are only now beginning to be recognized, and which appear to account for between one-half and three-quarters of all transcripts. At least 50%, and possibly the majority, of the human genome is transcribed. He fielded the hypothesis that RNAs derived from processed introns are involved in gene–gene communication and networking in real time in eukaryotic cells. The talk summarized the accumulating evidence

Copyright © 2003 John Wiley & Sons, Ltd.

that spliced RNA molecules are functional; introns comprise, on average, 95% of the primary sequence of protein-coding transcripts in humans, the numbers and size of introns and non-coding RNA correlates with developmental complexity, and some introns/non-coding RNAs are highly conserved.

The emphasis on systems biology was continued in the next session on expression arrays and networks. While there were many interesting presentations, the one that clearly stood out was that of **Eran Segal** (**Stanford University**; **www-cs.stanford.edu/~eran**) on 'Discovering molecular pathways from protein interaction and gene expression', which received the award for Best Student Paper of the meeting (more details of this work can be found at **http://bioinformatics.oupjournals.org/**). Segal presented results combining two *Saccharomyces cerevisiae* gene expression datasets with a protein interaction dataset and applying a unified probabilistic model to discover coherent functional groups and entire protein complexes. Later in the meeting, he presented further work extending these concepts in his talk, 'Discovery of transcriptional modules from DNA sequence and gene expression', which was awarded the Best Paper of the meeting.

At present, we have vast amounts of data sources, DNA sequence data, gene expression data and data from proteomic technologies such as yeast two-hybrid systems. The great challenge is how to use this data to better understand biological systems. On the second day of the meeting, these concepts were discussed in the keynote address of **Ron Shamir** (**Tel Aviv University**; **http://www.math.tau.ac.il/~rshamir/**), 'Reconstructing genetic networks'. His presentation emphasized the importance of developing computational methodologies that are rigorous, robust and realistic to help integrate the different datasets that are available. He emphasized the point that, as these datasets are highly heterogeneous, the scientific community should not expect any single 'killer application'. Tools that are developed will be tuned for a specific task. His group has implemented the CLICK tool and tested it on a variety of biological datasets, ranging from gene expression to cDNA oligo-fingerprinting to protein sequence similarity. CLICK was successfully used for gene clustering in functional genomics, finding motif sequences, tissue classification and more [18,7]. Other tools that his group has developed for the analysis of gene expression data include the new biclustering algorithm SAMBA (Statistical Algorithmic Method for Bicluster Analysis) and PRIMA (PRomoter Integration in Microarray Analysis), a program for finding transcription factors whose binding sites are enriched in a given set of promoters. By utilizing human genomic sequences and models for binding sites of known transcription factors, PRIMA identifies transcription factors whose binding sites are significantly over-represented in a given set of promoters. Another JAVA-based tool his group has developed to aid clustering and visualizing of gene expression data is EXPANDER (EXPression ANalyzer and DisplayER). This visualization tool includes an implementation of the new CLICK clustering algorithm, as well as for other popular clustering algorithms, such as K-means, self-organizing maps and hierarchical clustering. One of the important take-home messages of the entire meeting, particularly from the point of view of an experimental biologist like myself, which was highlighted by Shamir, is the importance of improved networking between those developing computing algorithms and experimentalists, to verify the relevance of newly developed tools. These methodologies are in their infancy and the long-term challenge is to encourage more research and effort to use these methodologies to make a dent in problems related to medicine and health.

This year's recipient of the ISCB Overton Prize was **Mr William James (Jim) Kent** (**UC Santa Cruz**; **http://www.cse.ucsc.edu/~kent**). This prestigious prize is awarded to a bioinformatician for outstanding accomplishment in the early phase of a career. His talk, entitled 'Patching and Painting the Human Genome', outlined the efforts that have gone into making the human genome more understandable to humans, i.e. providing visual representations of the human genome. Kent is best known as the researcher who 'saved' the human genome project. He wrote GigAssembler [10], a program that produced the first full working draft assembly of the human genome, the Human Genome Browser at UCSC (**http://genome.ucsc.edu** [11]), just before the company Celera was to present a complete draft of the human genome to the White House in 2000. This feat enabled the research community to keep human genomic data freely available in the public domain. Kent's talk summarized the goals of his work and introduced the bioinformatics tools he has built. He outlined the work

Copyright © 2003 John Wiley & Sons, Ltd. 

involved in developing tools such as: the Intronerator system for exploring the genome of *C. elegans* [12]; the program WABA, which was one of the first pair-hidden Markov models for the alignment of genomic DNA of two species; Improbiser, an expectation-maximization method to discover and cluster potential transcription factor binding sites; and the popular BLAT, which rapidly searches full genomes at both the DNA and protein levels [9]. As discussed by Kent, the UCSC browser is constantly evolving to include visualization of other genomes, such as mouse, and the goal of future research will be to provide tools that allow us to visualize multiple genomes more easily and will focus on integrating RNA data and allowing comparisons between genomes. The ultimate aim of these tools is to allow us to use genomic information more fruitfully.

This year the Society successfully introduced a new parallel stream to the ISMB program and the Tuesday afternoon was dedicated to short papers in parallel with meeting reports of the various SIGS. This new approach was well received by all attendees.

The third day of the meeting opened with a session on protein clustering, alignment and patterns and the morning concluded with a keynote from the recipient of the inaugural Senior Scientist Accomplishment Award, **David Sankoff** (**University of Ottawa**; **http://www.crm.umontreal.ca/cgi/qui?sankoff**). This new award was established in order to recognize a member of the computational biology community who is more than 12–15 years post-degree who has made major contributions to the field of computational biology through research, education, service, or a combination of the three. Sankoff received this award for the immense contributions he has made to computational biology during his career. Over the last 15 years his work has focused on the evolution of genomes as the result of chromosomal rearrangement processes [1,16]. He argued that the increase in large-scale genomic sequence data will give computational biologists the ability to compare theoretical work to experimental work! The scientific community now has many inventories of the random rearrangement and evolution that occur amongst different genomes, and now needs to use mathematical processes to look at these comparative maps/genomes, to get more realistic ideas about rates and overall tendencies in evolution [6].

The Wednesday afternoon session began with the next keynote of the meeting, 'Dynamic programming algorithms for haplotype block partitioning', delivered by **Michael Waterman** (**University of Southern California**; **http://www-hto.usc.edu/people/Waterman.html**). His lecture focused on using computational approaches to analyse molecular sequence data collected from human variation and single nucleotide polymorphisms (SNPS) [21,22]. The technology is now available to score large numbers of DNA variants (SNPs) in different individuals. The challenge is to be able to associate SNP data with different disease states when the problem suffers from excessive dimensionality. Waterman discussed the importance of developing a means to reduce the number of dimensions to the space of genotype classes in a biologically meaningful way. Linked SNPs are often statistically associated with one another (in 'linkage disequilibrium') and the number of distinct configurations of multiple tightly linked SNPs in a sample is often far lower than one would expect from independent sampling. These joint configurations, or haplotypes, might be a more biologically meaningful unit, since they represent sets of SNPs that co-occur in a population. Recently there has been much excitement over the idea that such haplotypes occur as blocks across the genome, as these blocks suggest that fewer distinct SNPs need to be scored to capture information about genotype identity. There is a need for formal analysis of this dimension reduction problem, for formal treatment of the hierarchical structure of haplotypes, and for consideration of the utility of these approaches toward meeting the end goal of finding genetic variants associated with complex disease.

Thursday was the last day of the meeting, and both keynote speakers once again gave presentations reminding us of the power of systematic approaches in science. **Yoshihide Hayashizaki** (**RIKEN Genomic Science Center**; **http://genome.gsc.riken.go.jp/index.html**) reviewed the amazing amount of work that has been accomplished in analysing the mouse transcriptome in his presentation, 'The dynamic eukaryotic transcriptome'. The RIKEN centre have just completed their mouse 'encyclopedia project', a map of the mouse transcriptome. This project further reinforced the importance of computer scientists and biologists working together, and with such a high level of collaboration it has been possible

Copyright © 2003 John Wiley & Sons, Ltd.

to publish the mouse genome and transcriptome together. Hayashizaki outlined the important principles behind the RIKEN transcriptome approach vs. the EST approach, which proved an invaluable aid to annotating the human genome. The RIKEN group decided that, as there was a saturation of human ESTs and as the transcriptome was more dynamic than the genome, it was more suitable for systematic analysis compared to the proteome. The secondary goal of the RIKEN mouse genome encyclopedia project was to develop a series of new and original technologies to aid investigation of transcriptomes [3,4]. The high-throughput sequencing technologies developed at RIKEN enabled them to sequence 40 000 samples per day. The FANTOM project led to functional annotation of 37 086 cDNAs, 20 487 protein-coding genes and 16 599 non-coding genes from the mouse [2]. This project was a tangible example of how international and interdisciplinary collaboration can lead to excellent science. Another of the goals of RIKEN was to develop a format by which the FANTOM clones (the genome encyclopedia) could be easily distributed to the scientific community. With something that seemed to be in the realms of science fiction, or else a scientific joke, Hayashizaki announced that a special issue of *Genome Research* had been produced — a test DNA book in which sample cDNA clones had been spotted on to water-soluble paper [8]. Incredibly, in this proposed DNA book, all the FANTOM clones will be clearly annotated, so that for each cDNA there would be a spot that could be punched out and, using PCR, a copy of the cDNA could be made. Thus, the DNA book provides an ingenious way for delivering DNA in a timely and cost-effective manner to the community.

Hayashizaki concluded his talk by reminding the audience that life science research in the twenty-first century is increasingly about connecting the genome to the phenome, and to facilitate this we will need to combine experimental approaches and computational approaches to allow new discoveries.

These comments served as a primer for **Sydney Brenner's** (**Salk Institute**; **http://www.salk.edu/faculty/faculty/details.php?id=7**) presentation, 'The evolution of genes and genomes'. Brenner provided a terrific message, reminding the audience of the simple concept that genes, including human genes, have not evolved independently from one another. By sequencing whole genomes we have gained much information but it needs to be considered in a completely fresh light, in a more systematic fashion which will involve computational biologists working in close conjunction with biologists. For a long time we have known that the mammalian genome is not homogenous in its GC composition, and that this base heterogeneity, or isochore structure, is correlated with other important genomic features, such as the insertion of repetitive elements and gene density [5]. Recently, it was shown that mutational rates are variable along the genome, pointing to a mosaic model of genome evolution. Large-scale comparisons of humans, rodents and frog genomes will be useful for learning about the evolution of mammalian genes and knowledge of gene chromosome position will allow visualization of the degree of mutational variation in the genome.

### Conclusions

This was my third and most scientifically enjoyable ISMB meeting to-date. This was due to the increased emphasis on systems biology and genomics throughout the entire program. For a molecular biologist and a newcomer to the field of bioinformatics like myself, ISMB once again provided a melting pot of opportunity to mingle with others new to the field, computer scientists and major players in the area, thus gaining a real feel for both the challenges and recent successes of the burgeoning bioinformatics scientific community. In addition, the meeting was a chance to renew acquaintances and meet new colleagues, and the experience reinvigorated my enthusiasm for computational biology and bioinformatics. More importantly, the take-home message of the meeting, for me, was the importance of providing many opportunities for computational biologists to work side-by-side with experimentalists to ensure that the work in both fields gives maximal outcomes in clinical diagnostics, human health and agriculture. These collaborations will ensure that the bioinformatics community comes up with robust and rigorous solutions to the key scientific challenges that genomic researchers face in the future.

By the end of the extremely lively and enthusiastic Glasgow 'ISMB 2004' presentation by **David Gilbert**, my bags were packed and I was ready to go. In another first, the next meeting will be held jointly with the European Conference on

Copyright © 2003 John Wiley & Sons, Ltd.

Computational Biology (ECCB) and in conjunction with Genes, Proteins and Computers VIII. Thus next year's ISMB meeting promises to deliver a program with a broader scope, but which will continue to focus on bringing the 'bio' back into bioinformatics. So, ISMB Glasgow 2004 in Scotland, all that rain and those castles can't wait!

### References

1. Blanchette M, Kunisawa T, Sankoff D. 1999. Gene order breakpoint evidence in animal mitochondrial phylogeny. *J Mol Evol* **49**(2): 193–203.
2. Bono H, Kasukawa T, Furuno M, Hayashizaki Y, Okazaki Y. 2003. FANTOM-DB: database of functional annotation of RIKEN mouse cDNA clones. *Seikagaku* **75**(2): 149–152.
3. Bono H, Yagi K, Kasukawa T, *et al.* 2003. Systematic expression profiling of the mouse transcriptome using RIKEN cDNA microarrays. *Genome Res* **13**(6B): 1318–1323.
4. Carninci P, Waki K, Shiraki T, *et al.* 2003. Targeting a complex transcriptome: the construction of the mouse full-length cDNA encyclopedia. *Genome Res* **13**(6B): 1273–1289.
5. Castresana J. 2002. Genes on human chromosome 19 show extreme divergence from the mouse orthologs and a high GC content. *Nucleic Acids Res* **30**(8): 1751–1756.
6. Eichler EE, Sankoff D. 2003. Structural dynamics of eukaryotic chromosome evolution. *Science* **301**(5634): 793–797.
7. Elkon R, Linhart C, Sharan R, Shamir R, Shiloh Y. 2003. Genome-wide *in silico* identification of transcriptional regulators controlling the cell cycle in human cells. *Genome Res* **13**(5): 773–780.
8. Kawai J, Hayashizaki Y. 2003. DNA book. *Genome Res* **13**(6B): 1488–1495.
9. Kent WJ. 2002. BLAT — the BLAST-like alignment tool. *Genome Res* **12**(4): 656–664.
10. Kent WJ, Haussler D. 2001. Assembly of the working draft of the human genome with GigAssembler. *Genome Res* **11**(9): 1541–1548.
11. Kent WJ, Sugnet CW, Furey TS, *et al.* 2002. The human genome browser at UCSC. *Genome Res* **12**(6): 996–1006.
12. Kent WJ, Zahler AM. 2000. The intronerator: exploring introns and alternative splicing in *Caenorhabditis elegans*. *Nucleic Acids Res* **28**(1): 91–93.
13. Lander ES, Linton LM, Birren B, *et al.* 2001. Initial sequencing and analysis of the human genome. *Nature* **409**(6822): 860–921.
14. Mattick JS. 2001. Non-coding RNAs: the architects of eukaryotic complexity. *EMBO Rep* **2**(11): 986–991.
15. Mattick JS, Gagen MJ. 2001. The evolution of controlled multitasked gene networks: the role of introns and other noncoding RNAs in the development of complex organisms. *Mol Biol Evol* **18**(9): 1611–1630.
16. Sankoff D. 2001. Gene and genome duplication. *Curr Opin Genet Dev* **11**(6): 681–684.
17. Seipel A, Haussler D. 2003. Combining phylogenetic and hidden Markov models in biosequence analysis. *Proceedings of the 7th Annual International Conference on Research in Computational Molecular Biology (RECOMB'2003).*
18. Sharan R, Shamir R. 2000. CLICK: a clustering algorithm with applications to gene expression analysis. *Proceedings of the International Conference on Intelligent Systems for Molecular Biology; ISMB* **8**: 307–316.
19. Thomas JW, Touchman JW, Blakesley RW, *et al.* 2003. Comparative analyses of multi-species sequences from targeted genomic regions. *Nature* **424**(6950): 788–793.
20. Waterston RH, Lindblad-Toh K, Birney E, *et al.* 2002. Initial sequencing and comparative analysis of the mouse genome. *Nature* **420**(6915): 520–562.
21. Zhang K, Deng M, Chen T, Waterman MS, Sun F. 2002. A dynamic programming algorithm for haplotype block partitioning. *Proc Natl Acad Sci USA* **99**(11): 7335–7339.
22. Zhang K, Sun F, Waterman MS, Chen T. 2003. Haplotype block partition with limited resources and applications to human chromosome 21 haplotype data. *Am J Hum Genet* **73**(1): 63–73.

Copyright © 2003 John Wiley & Sons, Ltd.
